# Impact of Probiotics on Dairy Production Efficiency

**DOI:** 10.3389/fmicb.2022.805963

**Published:** 2022-06-09

**Authors:** Kirankumar Nalla, Naresh Kumar Manda, Harmeet Singh Dhillon, Santosh R. Kanade, Namita Rokana, Matthias Hess, Anil Kumar Puniya

**Affiliations:** ^1^Department of Plant Science, School of Life Sciences, University of Hyderabad, Hyderabad, India; ^2^Department of Biosensors and Nanotechnology, CSIR-Institute of Microbial Technology, Chandigarh, India; ^3^Dairy Microbiology Division, ICAR-National Dairy Research Institute, Karnal, India; ^4^Department of Dairy Microbiology, College of Dairy Science and Technology, Guru Angad Dev Veterinary and Animal Sciences University, Ludhiana, India; ^5^Systems Microbiology and Natural Product Discovery Laboratory, Department of Animal Science, University of California, Davis, Davis, CA, United States

**Keywords:** probiotics, feed supplements, biotherapeutics, dairy production, gut microbiome

## Abstract

There has been growing interest on probiotics to enhance weight gain and disease resistance in young calves and to improve the milk yield in lactating animals by reducing the negative energy balance during the peak lactation period. While it has been well established that probiotics modulate the microbial community composition in the gastrointestinal tract, and a probiotic-mediated homeostasis in the rumen could improve feed conversation competence, volatile fatty acid production and nitrogen flow that enhances the milk composition as well as milk production, detailed changes on the molecular and metabolic level prompted by probiotic feed additives are still not understood. Moreover, as living biotherapeutic agents, probiotics have the potential to directly change the gene expression profile of animals by activating the signalling cascade in the host cells. Various direct and indirect components of probiotic approaches to improve the productivity of dairy animals are discussed in this review.

## Introduction

Livestock production is a dynamic sector, critical to satisfying the growing demand for animal-sourced products and dairy products specifically have become a significant source of income for farmers contributing to the growth of developing economies (Thornton, 2010; Tona, 2021). The increasing pressure of the global population, limited arable land, and climate change has led to the urgent need of advanced approaches to enhance cow health and productivity and to make dairy production sustainable in a rapidly changing environment (Britt et al., 2018). Despite the increasing demand, the dairy sector in low- and middle-income countries is still struggling with the challenge of low animal productivity. The use of natural and inexpensive probiotics-based supplements as alternatives to antibiotics to promote animal growth and health has increased in recent years in the livestock industry, especially since the use of antibiotics as growth promoters has been strictly regulated in many countries to limit the evolution and distribution of antibiotic resistance through the food system (Sharma et al., 2018a).

Probiotics are living non-pathogenic microbes and in many cases are also naturally present to some extent in the gastrointestinal tract. Over the years numerous bacteria and fungi have been identified as probiotics (Table 1) (Choct, 2009; Puniya et al., 2015; Markowiak and Ślizewska, 2018; Zommiti and Ferchichi, 2021). Supplementation with probiotics aids in maintaining gut microflora homeostasis, which improves feed conversion efficiency ultimately resulting in increased milk and meat production (Jinturkar et al., 2009; Maake et al., 2021; Mani et al., 2021). Furthermore, probiotics have also been reported to reduce levels of stress-related markers, such as cortisol (Zhang et al., 2016). Here we have outlined major effects of commonly used probiotics on dairy animals’ health, nutrition, and productivity.

**Table 1:** Probiotic fungi and bacteria for ruminants.

**Organism**	**Species**	**Reference**
**Fungi**	*Aspergillus oryzae* *C. rugosa, C. pararugosa* *C. ethanolica, Magnusiomyces capitatus* *Debaryomyces hansenii* *Saccharomyces boulardii* *S. cerevisiae* *Pichia kudriavzevii, Candida tropicalis, Galactomyces* sp.	Sucu et al., 2018 Fernandes et al., 2019 Angulo et al., 2019 Santos et al., 2021 Shakira et al., 2018 Suntara et al., 2021a
**Bacteria (Gram-positive)**		
*Lactobacillus*	*Lactobacillus acidophilus* *L. alimentarius* *L. amylorvous* *L. animalis* *L. casei* *L. mucosae* *L. plantarum* *L. reuteri, L. johnsonii, L. amylovorus* *L. rhamnosus* *L. salivarius* *L. sporogenes* *L. sakei* *Ligilactobacillus salivarius*	Sharma et al., 2018b Apas et al., 2014 Maldonado et al., 2012 Ayala et al., 2018 Ayala-Monter et al., 2019 Royan et al., 2021 Izuddin et al., 2019 Fernandez et al., 2018 Maake et al., 2021 Stefańska et al. (2021) Shreedhar et al., 2016 Sasazaki et al., 2020 Varada et al., 2022
**Other lactic acid bacteria**	*Lactococcus lactis* *Streptococcus bovis* *Enterococcus faecalis* *E. faecium* *Pediococcus acidilactici* *P. pentosaceus* *Propriobacterium freudenreichii*	Armas et al., 2017 Aphale et al., 2019 Maake et al., 2021 Marcinakova et al., 2004 Reddy et al., 2011 Ladha and Jeevaratnam, 2018 Vasconcelos et al., 2008
*Bifido-bacterium*	*Bifidobacterium bifidum* *B. longum, B. animalis* *B. pseudolongum* *B. ruminantium*	Apas et al., 2014 Bunesova et al., 2012 Maake et al., 2021 Vlkova et al., 2009
*Bacillus*	*Bacillus amyloliquefaciens* *B. licheniformis* *B. subtilis,* *B. subtilis natto* *B. toyonensis*	Schofield et al., 2018 Devyatkin et al., 2021 Devyatkin et al., 2021 Chang et al., 2021 Santos et al., 2021
**Other**	*Paenibacillus* sp.	Latham et al., 2018
**Bacteria (Gram-negative)**		
	*Butyrivibrio fibrisolvens* *E. coli* *E. coli* Nissle 1917 *Megasphaera elsdenii* *Prevotella bryantii*	Fukuda et al., 2006 Tkalcic et al., 2003 Von Buenau et al., 2005 Carey et al., 2021 Chiquette et al., 2012

## Impact of Probiotic Microorganisms on Animals’ Welfare

Dairy production is considered to be among the top industrial sectors and global milk production reached nearly 906 million tonnes in 2020 (FAO, 2021). Thereby, any undesirable shortcoming in milk production caused by disease or malnutrition in lactating animals could cause substantial economic losses and a heavy disease burden in the livestock chain could also lead to public health threats, such as the emergence of antibiotic resistance (Sharma et al., 2018a). Application of probiotics for maintaining the general health, immunity and nutritional requirement of dairy animals and other livestock could provide a sustainable solution to mitigate some of these problems.

**Figure 1:**
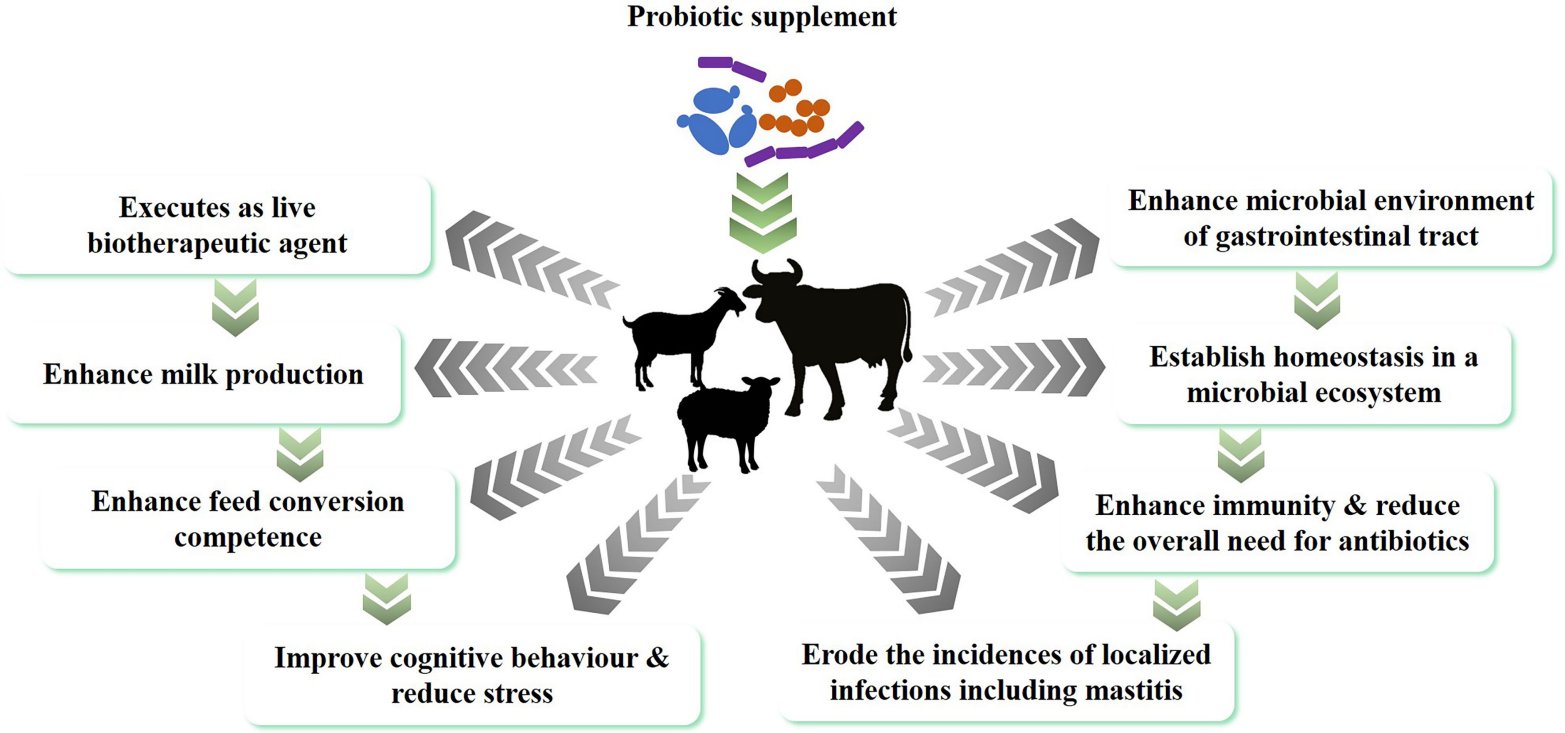
Impact of probiotics on the general health of dairy animals.

Probiotics are live beneficial bacteria that, when administered in adequate amounts, confer a health benefit on the host (Hill et al., 2014), often by colonizing the gastrointestinal tract and supporting the native microflora that is already established in the animal’s digestive system. Indirectly, probiotics can also support mucosal immunity by promoting the beneficial mucosal microflora and preventing colonization of the mucosa by pathogens (Uyeno et al., 2015). In addition, the general health benefits of probiotics in the digestive system of ruminants also includes the control of acidosis, digestive comfort, reducing methanogenesis, promoting the growth of rumen and intestinal epithelium, increased nutrient uptake, and an increased feed conversion ratio (Abd El-Tawab et al., 2016; Retta, 2016). Reduced inflammation markers in the host are also linked to the establishment of probiotic mediated intestinal homeostasis that strengthens the animal’s natural defense mechanism and overall health (Figure 1). The different routes through which probiotics can benefit the general health of animals are summarized in the following sections.

### Gut and Rumen Microflora Homeostasis

The microbiome of the gastrointestinal tract plays a critical role in the well-being of the host animal due to its ability to closely regulate the components of both innate and adaptive immunity. A healthy microflora keeps animal healthy by maintaining immunological homeostasis and removing pathogenic microorganisms (Wu and Wu, 2012; Butel, 2014). The rumen microbiome has also been proven to significantly impact the development of native and systemic immune components (Wu and Wu, 2012). The gut microbial ecosystem is made up of a diverse mix of bacteria, anaerobic fungi, and ciliated protozoa (Chaucheyras-Durand and Durand, 2010). Therefore, probiotics are primarily used to establish homeostasis conditions within the gut and rumen microflora by supporting the population of beneficial microbial species (Xu et al., 2017). Probiotics specifically enhance the relative abundance of beneficial genera that help to prevent pathogenic invasion into the gastrointestinal tract. For instance, Fernández-Ciganda et al. (2021) reported that supplementing two probiotic strains, *Lactobacillus johnsonii* TP1.6 and *Lactobacillus reuteri* TP1.3B, to young calves increased the abundance of beneficial taxa including *Bifidobacterium* and *Akkermansia* in the intestine. Similarly, *Bacillus amyloliquefaciens* fsznc-06 and *Bacillus pumilus* fsznc-09 enhanced the relative richness of potentially beneficial bacteria in the rumen and intestine of weanling goats (Zhang et al., 2020). This beneficial shift was also correlated with enhanced ruminal papilla and small intestinal villus growth of the studied animals. In another study, the effect of a multi-strain probiotic product containing *Bifidobacterium animalis*, *Lactobacillus casei*, *Streptococcus faecalis*, and *Bacillus cerevisiae* on general health and the fecal bacterial composition of Holstein’s calves were investigated and it was found that although the probiotic supplements did not affect the overall fecal microflora at the phylum level, the relative abundance of beneficial *Prevotella* increased while abundance of the opportunistic pathogens *Dorea* decreased (Guo et al., 2022), suggesting that this probiotic supplements supported an enhanced defence mechanism of the rumen and the intestine, potentially reducing the risk of colonization by pathogens.

### Digestion Efficiency

Lignocellulosic plant fibers, which are significant components of the ruminant diet, require the symbiotic activity of diverse microorganisms to hydrolyze and extract nutrients from it. The rumen microbial community is a diverse blend of taxa from prokaryotic bacteria and archaea as well as eukaryotic fungi and protozoa. During the preweaning stage of life, the relative number of beneficial genera of Bacteroidetes, Firmicutes, Proteobacteria remains dominant, followed by Elusimicrobia, Fibrobacteres, *Bifidobacterium*, Tenericutes and Verrucomicrobia (Meale et al., 2016). However, at the later stage of life, change in feed shifts the microbial composition by significantly increasing the population of Proteobacteria and decreasing the number of Bacteroidetes, Elusimicrobia, Fibrobacteres, Tenericutes, and Verrucomicrobia in dairy calves (Meale et al., 2017). The alteration in the composition of the commensal microbial population could significantly influence the fermentation, digestion and nutrient uptake from the diet. Likewise, Zhang et al. (2016) suggested that the ratio of Bacteroidetes, Firmicutes, and Proteobacteria could improve the feed conversion efficiency and reduce enteric methane production from rumen fermentation process. The role of probiotics to improve the digestive efficiency and feed conversion ratio in dairy animals has also been observed by the researchers. A preliminary *in vitro* study suggested that probiotics could improve the rumen organic matter digestibility as well as reduce total gas and methane production (Ellis et al., 2016). Kondrashova et al. (2020) studied the possible effect of the combination of *Bifidobacterium longum*, *Bifidobacterium adolescentis* and *Lactobacillus acidophilus* with vanillin in rumen fluid on the metabolic state of rumen microflora. *In vitro* evaluation suggested that the combination of vanillin with these three probiotic strains increase the digestibility of the dry feed. Besides, probiotics could simultaneously affect the rumen fermentation and rumen microbial community structure to improve the digestive metabolism. For instance, the activity of *Bacillus subtilis natto* reportedly improved the concentrations of ammonia nitrogen, microbial protein and volatile fatty acids (VFA) in rumen fluid connected to the change in 18 genera of rumen fluid (Chang et al., 2021).

Probiotic-mediated mechanisms to improve the digestive efficiency also includes the control of diet-induced acidosis and detoxification of harmful metabolites. Lactating animals are usually kept on concentrated grain-rich diets that increase the risk of rumen acidosis due to access accumulation of organic acid and VFAs exceeding the buffering capacity of rumen. Rumen acidosis is one of the most prevalent digestive disorders seen in cattle, with ranging from sub-acute to acute severity (Nagaraja and Titgemeyer, 2007). The negative impact of rumen acidosis can also lead to additional severe health problems to the other organs, such as laminitis and liver swelling (Enemark, 2008). Earlier studies in this line have shown that live yeast can help to stabilize rumen pH and reduce susceptibility to acidosis in dairy animals (Chaucheyras-Durand et al., 2008; Marden et al., 2008; Maamouri and Ben Salem, 2021).

Besides concentrated grain, many dairy animals are often kept on a diet that is naturally high in nitrate to reduce enteric methane production. Microbial conversion of nitrate to ammonium also yields nitrite as toxic intermediate and accumulation of nitrite can cause severe methemoglobinemia and acute toxicosis in ruminants. Effects of nitrite toxicosis may also be subacute or chronic and can include delayed growth, decreased milk production, vitamin A deficiency, minor transitory goitrogenic effects, abortion, fetotoxicity, and increased susceptibility to infection. Other sources of disproportionally high nitrate concentration are run-off water or shallow wells in regions where large amounts of fertilizers are applied to the surface (Hall, 2018). Latham et al. (2019) noted that administration of the nitrite-metabolizing bacterium *Paenibacillus fortis* to the rumen could promote nitrite metabolism and thus remove the toxic nitrite produced from the rumen. Hence, the use of probiotic-supplement could enhance the digestion efficiency and reduce the accumulation of toxic metabolites in the animals’ digestive tract.

### Intestinal Antimicrobial Activity

**Figure 2:**
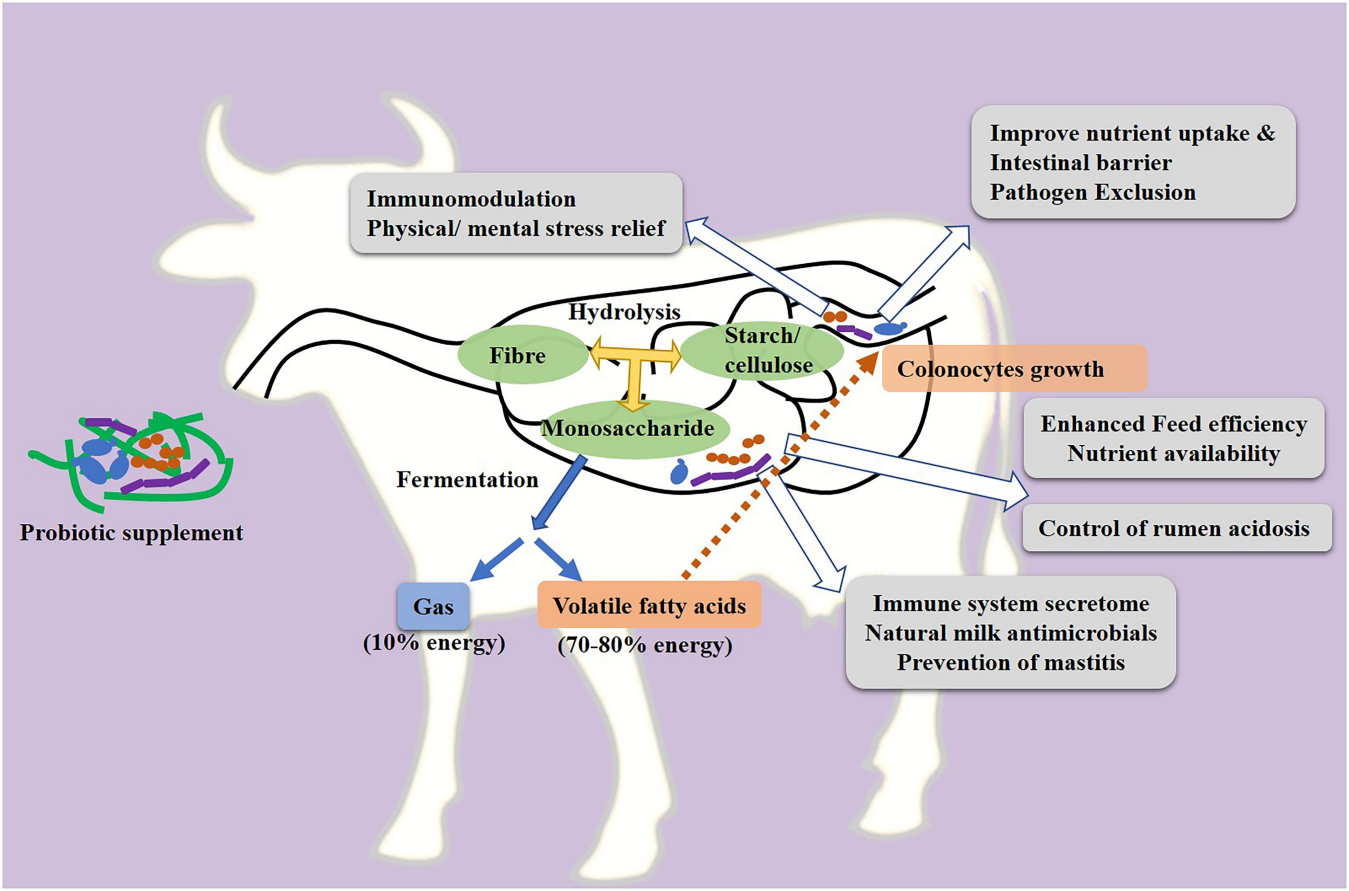
Probiotic activity to enhance the growth and general health of dairy animals. Yellow arrows in the figure indicate enzymatic digestion of feed in the gut; blue arrows indicate microbial fermentation of feed-derived monosaccharides; white arrows indicate the different beneficial mechanisms of probiotics, and the dotted red arrow indicates the VFA-mediated growth promotion of colonocytes.

A major cause of reduced milk productivity is animal sickness during the lactation period (Carvalho et al., 2019). Different studies with *in vitro* models have proven that probiotic microorganisms which naturally colonize the digestive tract of the host animal show strong antimicrobial activity against enteric pathogens (Adeniyi et al., 2015; Lin et al., 2020). The antimicrobial activity of probiotics against the enteric pathogens suggests their utility as an effective biotherapeutic to prevent related gastrointestinal infections (Prabhurajeshwar and Chandrakanth, 2019). Specifically these native probiotics support the intestinal epithelial barrier by enhancing the expression of barrier function components (Rokana et al., 2016; Bron et al., 2017) and prevent the enteric infections in the host (Lucey et al., 2021). Many of the known *Lactobacillus* probiotic strains produce metabolites, such as diacetyl, acetoin, hydrogen peroxide, and bacteriocins, which prevent the growth of pathogens and assist in defence mechanisms that are involved keeping infections at bay (Osuntoki and Korie, 2010; Pyar and Peh, 2014). For example, *Pediococcus pentosaceus* strains LJR1, LJR5, and LJR9 isolated from the rumen fluid of a healthy goat exhibited antibacterial activity against a broad spectrum of foodborne pathogens such as *Listeria monocytogenes*, *Escherichia coli* and *Streptococcus pyrogenes* (Ladha and Jeevaratnam, 2018). Probiotics could also prevent infection by competing with pathogens for attachment to the intestinal epithelium (Rokana et al., 2017; Figure 2).

The preventive mode of probiotic action against different types of infections is also currently being investigated. For instance, parasite-borne diseases, in addition to bacterial infections, are prevalent in dairy animals. The beneficial effects of probiotics on the recovery from parasite infections, such as helminths (e.g., *Trichuris, Ascaris*), *Eimeria* and *Cryptosporidium*, have been proven in several studies using various animal models (Travers et al., 2011). Furthermore, numerous farm-level case studies in poultry sector have shown that the use of probiotics (including *Pediococcus*, *Lactobacillus*, *Enterococcus*, *Bifidobacterium*, and *Bacillus* species) improved the host resistance to *Eimeria*-borne coccidiosis (Dalloul et al., 2003; Lee et al., 2007; Giannenas et al., 2012; Abdelrahman et al., 2014). Similarly, researchers have reviewed the possible approach of manipulation of ruminant gut microbiome to control the parasite infection in dairy animals (Cortés et al., 2020). Ramírez et al. (2021) have attepted to identify the compositional change in intestinal microflora of *Fasciola hepatica* infected Holstein cows. The study revealed reduced diversity in prokaryotic and eukaryotic microorganisms and a specific decrease in the relative abundance of *Bacteroidetes* and fungi Ascomycota in *Fasciola* infected cows. Earlier studies on probiotic-mediated gut microbiome manipulation suggest that probiotics could be used as an alternative strategy to ameliorate the helminth infections in dairy animals. However, more clinical research focusing on the probiotic mechanism in the presence of parasite infection is needed to determine their efficacy in this area.

**Figure 3:**
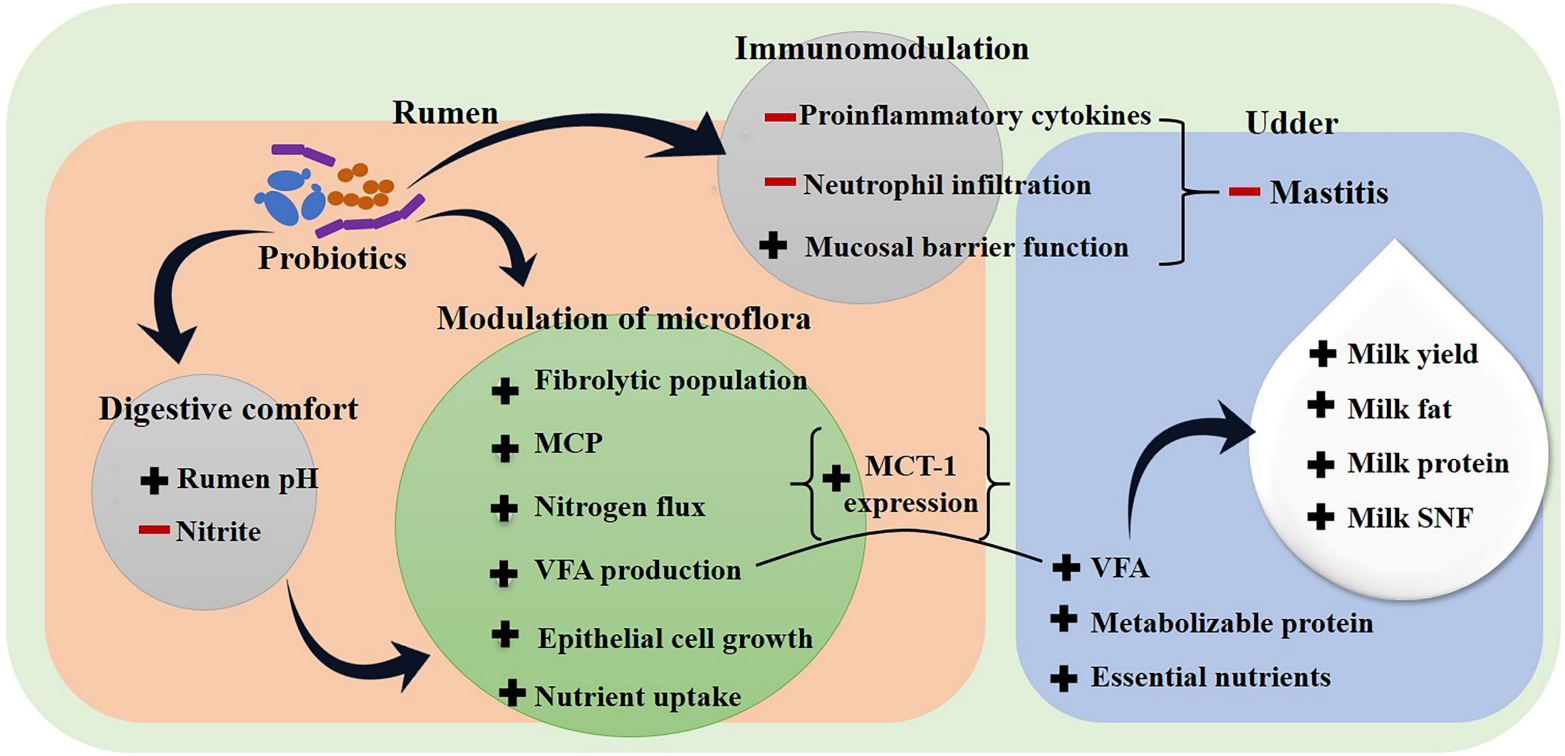
Effect of probiotics to improve milk productivity in dairy animals. (**−**) Sign indicates inhibition, (**+**) sign indicates enhancement, MCP: microbial crude protein, SNF: slid not fat, VFA: volatile fatty acid.

The antiviral efficacy of probiotics against viruses that cause intestinal, urogenital and respiratory infections have also been successfully demonstrated (Arena et al., 2018). Probiotics may directly interact with viruses, inhibit them using antiviral metabolites or they may indirectly influence the host immune system against viral pathogenesis (Al Kassaa et al., 2014; Drider et al., 2016). The live cells of probiotics use adsorptive, trapping mechanisms or produce hydrogen peroxide, lactic acid and antimicrobial peptides to inactivate target viruses (Al Kassaa et al., 2014). Villena et al. (2018) have reviewed that these beneficial microbes could also stimulate the antiviral innate immune response by activating the expression of antiviral pattern recognition receptors in host cells that subsequently induce the secretion of nuclear factor κB pathway-related proinflammatory cytokines and chemokines. The activation of the nuclear factor κB dependent pathway help to reduce the severity of viral infection in the animal host (Villena et al., 2018). Even though these studies are limited to *in vitro* cell line models or small animals, the results are promising and comparable effects are anticipated to occur in extended *in situ* experiments. In addition to enteric, parasite and viral infections, mastitis is a highly prevalent disease in lactating dairy animals that causes a substantial economic loss to milk producers (Bhakat et al., 2020). Several probiotics administered orally or locally have shown their potential to reduce mastitis infection by modulating the intestinal microbial composition, immunological responses and by enhancing the epithelial barrier function (Figures 2 and 3; Tanbayeva et al., 2016; Armas et al., 2017; Rainard and Foucras, 2018; Pellegrino et al., 2019; Steinberg et al., 2021). Different types of preventive mechanisms have been proposed with multiple types of probiotics. For instance, *Saccharomyces cerevisiae* and *Lactococcus* species reduced the mastitis-related inflammation by lowering serum concentration of tumor necrosis factor-α (TNF-α), Interleukin-6 (IL-6), and IL-1β in dairy cows (Gao et al., 2020). Both probiotics also inhibited the neutrophil infiltration indicator, i.e., milk myeloperoxidase and a mastitis indicator, i.e., N-Acetyl-β-d-Glucosaminidase activity in milk suggesting a decline in mastitis related damage to mammary cells. Likewise, an *in vitro* study demonstrated the immunomodulatory potential of probiotic *L. casei* BL23 on bovine mammary epithelial cells infected with mastitis causing *Staphylococcus aureus* (Souza et al., 2018). The work indicated the indirect mechanism of probiotic action against mastitis pathogens. *L. casei* BL23 decreased the expression of several pro-inflammatory cytokines, including interleukins IL-6, IL-8, IL-1α and IL-1β and TNF-α in *S. aureus*-stimulated mammary epithelial cells. Interestingly, probiotics also slightly improved the expression of antimicrobial peptides and defensin β in host cells. Investigation of the entire mechanism showed that probiotic *L. casei* BL23 has helped to recover from mastitis infection by reducing inflammation and strengthening the cellular protection mechanism of bovine mammary cells. A study on heat-inactivated *Lactobacillus gasseri* LA806 to counteract *S. aureus* and *E. coli* infection in an *in vitro* model of bovine mastitis suggested that even inactivated probiotic cells could carry immunomodulatory properties (Blanchet et al., 2021). Observations recorded from these studies suggest that probiotics could modulate the host cell defense mechanism using the cell receptor-mediated channel.

### Stress Management *via* Modulation of the Gut-Brain Axis

The connection between intestinal microflora and the general health of the host has been well established and growing evidence suggests that the gut microbiome also impacts brain operation and cognitive behavior (Desbonnet et al., 2015) (Figure 2). Some important neurotransmitters related to mood regulation, i.e., serotonin, dopamine and aminobutyric acid, are produced in both gut and brain (Cheng et al., 2019). In addition, studies have supported the gut-brain axis theory by identifying the connection between the vagus nerve of the parasympathetic nervous system and gut microbiome (Bonaz et al., 2018). The functions of the vagus nerve include communication between the brain and intestinal epithelium that coordinates the individual’s adaptive response to environmental or physiological stress (Breit et al., 2018). The stress signals through the vagus nerve activate the pituitary–adrenal axis and a neural and hormonal communication that influence the activities of intestinal epithelial and immune cells (Breit et al., 2018). Studies have shown that the gut microbiome could influence the vagus nerve mediated effects using endocrine, immune or neural mechanisms (Fülling et al., 2019). The variety of the gut microbiome has been proven to regulate cognitive behavior by stimulating the synthesis of essential neurotrans-modulators (i.e., tryptophan and neuropeptides) in rodents (Desbonnet et al., 2015). The ability of probiotics to restore gut homeostasis has also been linked to reduced anxiety alleviating stress level in individuals (Scott et al., 2013; Zhang et al., 2019). A small number of investigations, involving farm animals, have revealed that the gut microbiome can modulate stress level in dairy animals in a similar way. Kelsey and Colpoys (2018) discovered that feeding probiotics can reduce stress-related behavior in weaned and developing calves. Based on these findings, probiotics may be used to help dairy animals to cope with stress behavior, but additional studies will be required to define the extent of how probiotics might facilitate improved stress response.

## Probiotics to Promote the Growth of Dairy Animals

Emergence and manifestation of antibiotic resistance in the food chain has prompted a search for alternatives to antibiotics that have growth-promoting effects on livestock. Since the diversity of the rumen microbiome is closely related to the animal’s ability to acquire and assimilate nutrients, ideal growth promoters would have only a negligible impact on the animal’s natural microbiome, while enhancing the animal’s growth, well-being and reproduction (Breves et al., 2000; Mostafa et al., 2014). Many animal probiotics have been shown to improve feed efficiency (Moreira et al., 2019), growth performance (Tripathi and Karim, 2010; Didarkhah and Bashtani, 2018), nitrogen retention (Schofield et al., 2018) and also reduced the risk of intestinal infections (Aldana et al., 2009; Tripathi and Karim, 2010; Signorini et al., 2012; Didarkhah and Bashtani, 2018).

Primarily, probiotics could improve the rumen and intestinal epithelial cells growth that enhance nutrition uptake capacity. Probiotics implement these beneficial effects by improving the production of VFAs that act as growth promoters for epithelial cells (Figure 2). An investigation on reweaning calves by Stefańska et al. (2021) explains the possible mechanism behind it. The calves received a multi strain probiotics feed additive containing *Lactobacillus casei* PKM B/00103, *Lactobacillus salivarius* PKM B/00102 and *Lactobacillus sakei* PKM B/00101 that improved ruminal fermentation and increased the concentration of total VFA, propionate, butyrate and the ratio of butyrate to valerate. The change in VFA profile further enhanced the total dry matter intake and growth of reweaning calves. Another interesting study on growth delayed calves by Du et al. (2018) has also presented similar observations. Supplementation of probiotics *B. amyloliquefaciens* and *B. subtilis* improved feed intake, energy and short-chain fatty acid production by increasing the number of intestinal fiber degrading bacteria, including *Proteobacteria*, *Rhodospirillaceae*, *Campylobacterales*, and *Butyricimonas*. Additionally, probiotic supplement improved the growth hormone/insulin-like grows factor-I ratio that enhanced body weights of growth delayed calves. Comparable observations are also recorded in sheep and lambs by Devyatkin et al. (2021). They found that supplementation of a commercial probiotics preparation Enzimsporin (consisting of *Bacillus subtilis* B-2998D, B-3057D, and *Bacillus licheniformis* B-2999D) increased body weight gain of sheep and lambs. The growth-promoting effect was related to the increasing intestinal *Lactobacillus* and *Bifidobacterium* population and decreasing *E. coli*, *Enterococcus*, and yeast in the fecal contents.

The growth promotion of dairy animals is also related to the nutrition extraction and retention from the provided feed. Schofield et al. (2018) found that probiotic *Bacillus amyloliquefaciens* H57 could benefit the ruminant host by increasing nitrogen retention, feed intake, weight gain and reducing the methane emission in ruminants (Table 2). The probiotic indirectly induced these effects by increasing the proliferation of plant fiber digesting *Prevotella* species and *Roseburia faecis* in the rumen. Likewise, Chen et al. (2021) observed the enhanced average daily gain in ruminant receiving feed supplemented with a combination of three probiotics, *Bacillus licheniformis*, *Bacillus subtilis* and *Lactobacillus plantarum* blended with a Chinese medicine polysaccharide. The probiotic effect was mediated by increasing the relative abundance of *Fibrobacteria* that promoted rumen protein fermentation. Probiotics could also target fibrolytic eukaryotic commensal microflora to improve nitrogen utilization from the roughage. Phesatcha et al. (2022) have determined the influence of adding *Saccharomyces cerevisiae* to the diet in cattle. The outcomes of the study revealed that yeast supplementation increased the nutritional digestibility and efficiency of microbial protein nitrogen production and nitrogen absorption while increasing fibrolytic fungi population and lowering the protozoa population. These results suggest that probiotics can improve the nutrition availability and physical growth that support the productivity of dairy animals.

**Table 2:** Commercial probiotic oral supplements that demonstrated specific health benefits in dairy animals.

Commercial probiotic supplement	Strains/probiotics	Host animal	Recommended dose	Probiotic effect	Reference(s)
Bacterial probiotics (Miyarisan Pharmaceutical Co., Ltd.)	*L. plantarum* 220*, Enterococcus faecium* 26 and *Clostridium butyricum* Miyari	Cattle	Daily single dose of 20, 50 and 100 g	Improved rumen pH in cattle with experimentally induced sub-acute rumen acidosis receiving 20 or 50 g probiotic dose	Goto et al., 2016
FASTtrack microbial pack (Conklin)	*Saccharomyces cerevisiae* and dried fermentation product of *E. faecium* and *Lactobacillus acidophilus*; fermentation extract of *Aspergillus oryzae* and *Bacillus subtilis*	Lactating cows	Daily 1 oz. administration with water	Impact on expression of immunity and homeostasis related markers	Adjei-Fremah et al. (2018)
Huijia′s *Clostridium butyricum* (Huijia Biotechnology Co. Ltd.)	*C. butyricum*	Goats	10^8^ CFU/g in basal diet	Improved rumen environment by decreased oxidation–reduction potential and increased pH	Cai et al., 2021
MultiBio 3PS (BiOWiSH Technologies Inc.)	*L. plantarum, Pediococcus acidilactici, P. pentosaceus, B. subtilis*	New-born calves	Pallet of 0.12 g/day and 1.2 g/day	High concentration improved calf immunity, serum antioxidative capacity and altered rumen fermentation	Wang et al., 2022
PrimaLac (Star-Labs)	*L. acidophilus, L. casei, Bifidobacterium thermophilum* and *E. faecium*	Lactating ewes	2 g mixed with concentrate feed	Positively affected milk yield and its components	Kafilzadeh et al., 2019
Revive™ (Partnar Animal Health)	*P. acidilactici*, *E. faecium*, *L. acidophilus*, *L. casei*, *Bifidobacterium bifidum*	Calves	4 g bolus of probiotics daily for 4 consecutive days	Reduced the duration of diarrhoea at small magnitude but did not show effect on the growth of calves	Renaud et al., 2019

## Probiotics to Improve the Productivity of Dairy Animals

In previous sections, we have discussed that the benefits of probiotic-based feed supplements on growth improvement are mediated by the change in the rumen microbiome composition that improved digestion and fermentation processes and ultimately increased nutrient bioavailability to the animal. Dairy nutritionists and researchers in this field also attempted to explore whether the similar impact of probiotics is extended to the milk productivity of the lactating animal. Studies have demonstrated that probiotic supplementation also improve the quality and quantity of milk production (Figure 3; Desnoyers et al., 2009; Xu et al., 2017). The role of probiotics in enhancing the milk production in dairy animals could be divided in two different modes.

The first is an indirect mechanism, where probiotics improve milk production by modulating the digestive metabolism, nutrient availability and uptake in the intestine. For example, So et al. (2021) found that *L. casei* TH14 had a positive effect on milk yield as well as other physiological factors such as digestibility of dry matter and fiber, gross energy, metabolic energy intake, blood glucose, and total VFAs, while suppressing somatic cell count in milk. The similar finding is corroborated in several studies where supplementing dairy animals with probiotics improved milk production without affecting the basic nutritional makeup of the milk (Moallem et al., 2009; Peng et al., 2012).

In addition, researchers have also recorded the qualitative improvement in milk production using probiotic interventions. A study conducted on lactating Holstein cows by Suntara et al. (2021b) presented an interesting fact that administration of animal feed prepared with Crabtree-negative yeast (*P. kudriavzevii* KKU20 and *C. tropicalis* KKU20) increased the milk protein content. The reason was connected to the increased beneficial microbial population in the rumen, which enhanced the amount of microbial crude protein. Similar observations on ruminal microbial crude protein mediated increased milk and milk protein yield were reported by Xie et al. (2019). The authors also comprehended the mechanism and explained that microbial crude protein is synthesized by microorganisms of the gut that significantly impact the quantity and quality of metabolizable protein to be absorbed in the intestine. The metabolizable protein subsequently converts to milk protein and influences milk yield. Ma et al. (2020) also noted the effects of *Saccharomyces cerevisiae*, *Bacillus subtilis* and *Enterococcus faecalis* on milk production and milk composition in lactating goats by increasing milk fat, protein and total solid percentage in milk. Also, Sun et al. (2021) assessed the effects of *Saccharomyces cerevisiae* on milk quality of dairy cows and reported that cows receiving probiotic supplementation produced more milk with higher fat content. Findings also included improved rumen pH, acetic and propionic ratio, indicating the influence of probiotics on increased milk fat content. Because after absorption, VFAs serve as precursors for milk synthesis (Figure 3).

The second mode is probiotic-mediated modulation of the gene expression profile of lactating animal. A direct mechanism of probiotic mediated modulation of gene expression in the dairy animal has been observed by Izuddin et al. (2019). The study was conducted on newly-weaned lambs receiving spent culture supernatant (i.e., postbiotic) of probiotic *L. plantarum* RG14. In addition to increasing the population of fiber degrading bacteria and decreasing the relative population of methanogens and protozoa in the rumen, postbiotics increased the gene expression of hepatic Insulin-like growth factor-1 and ruminal monocarboxylate transporter-1 (MCT-1). Growth factor-1 is a mediator of growth promoter hormone, whereas MCT-1 is associated with the rumen epithelium membrane transport system. The study does not present the evident correlation between milk productivity and probiotic mediated gene expression modulation. Yet, presumably, the increased expression of MCT-1 in lactating animals could induce higher uptake of VFA from rumen epithelium, resulting in improved milk production. Further identification of the active cellular components of probiotics that impact the gene expression profile related to milk production could open novel ways concerning the dairy industry.

Probiotics implement a beneficial impact on dairy animals’ growth and milk production efficiency. The underlying mechanism is chiefly connected with the management of the rumen and intestinal microbial community. The probiotic mediated microbial homeostasis favors the positive nutritional balance and strengthens the general health of the lactating animal. These beneficial microorganisms propose a natural, consequence free approach to boost the dairy production.

## Outlook

Besides general health advantages, probiotics could also serve as a potential biological source to improve the productivity of dairy animals. The natural mechanisms of probiotics include reducing disease burden, modulation of rumen metabolism and modulation of host gene expression. Collectively, it can be stated that the addition of probiotics in feed may enhance the yield of milk and improve the compositional quality of milk in lactating animals. However, the effects of the probiotics are influenced heavily by the strain variation, diet and host physiological state. Hence, developing new intervention strategies using probiotics or active molecular components from probiotics will be worth exploring.

## Author Contributions

KN, NKM, and NR did literature search and prepared the draft. HD participated in preparation of manuscript. SK, MH, and AKP reviewed and edited the manuscript. All the authors contributed to the article and approved the submission.

## Conflict of Interest

The authors declare that the research was conducted in the absence of any commercial or financial relationships that could be construed as a potential conflict of interest.

## Publisher’s Note

All claims expressed in this article are solely those of the authors and do not necessarily represent those of their affiliated organizations, or those of the publisher, the editors and the reviewers. Any product that may be evaluated in this article, or claim that may be made by its manufacturer, is not guaranteed or endorsed by the publisher.
